# Trajectories of Maternal and Paternal Internalizing Symptoms from Pregnancy to 2 Years Postpartum: Identifying Modifiable Risk and Protective Factors

**DOI:** 10.1155/2024/5164261

**Published:** 2024-07-30

**Authors:** Lauren M. Laifer, Erin L. Ramsdell, Sara M. Stasik-O'Brien, Rachel C. B. Martin, Rebecca L. Brock

**Affiliations:** ^1^Department of Psychology, University of Nebraska-Lincoln, Lincoln, NE, USA; ^2^Department of Psychiatry, Michigan Medicine, Ann Arbor, MI, USA; ^3^Department of Psychology, Carthage College, Kenosha, WI, Tunisia

## Abstract

There is an increased risk for depression and anxiety across the perinatal period (i.e., spanning pregnancy and the first year postpartum); however, limited research has examined elevations in core negative affectivity underlying internalizing disorders more broadly. The current study sought to characterize trajectories of core internalizing problems among both mothers and fathers across the perinatal period and explored whether modifiable risk and protective factors buffered risk for elevated symptoms during this key developmental transition. A community sample of mixed-sex couples (*N* = 159) completed assessments during pregnancy and at four postpartum timepoints. Using growth mixture modeling, we found that 21.2% of mothers demonstrated clinical elevations in core internalizing symptoms that persisted up to 2 years postpartum. In contrast, 7.8% of fathers demonstrated clinical elevations in core internalizing symptoms across this period, with an additional 29.0% of fathers demonstrating subthreshold symptom elevations. Concerns related to pregnancy and childbirth and paternal (partner) internalizing problems during pregnancy conferred risk for elevated symptoms in mothers, whereas psychological flexibility, emotional intimacy, and the quality of received support were identified as protective factors for fathers. Results highlight the importance of repeated screening for internalizing problems and suggest that promoting a strong interparental relationship is critical for emotional health and well-being across the perinatal period.

## 1. Introduction

Pregnancy and childbirth represent a critical developmental transition within the family system that is often characterized by both excitement [1, 2] and considerable stress [3]. The psychosocial stressors occurring across this transition may be exacerbated by the biological, social, and psychological changes that occur throughout pregnancy [4]. Pregnancy, therefore, poses a significant risk for parental psychopathology, particularly internalizing problems (i.e., depression and anxiety [5, 6, 7]). Heightened stress and psychopathology during pregnancy, in turn, are associated with negative consequences for parent and infant health and well-being during the postpartum period [8, 9]. In addition to being a period of vulnerability for psychopathology, pregnancy simultaneously offers a critical opportunity for intervention [10, 11]. Thus, early identification of parental internalizing problems, accompanied by evidence-based psychological interventions, is of particular importance for promoting individual and family well-being across this high-risk period.

A robust body of research demonstrates elevated risk for maternal depression across the perinatal period (i.e., spanning pregnancy and the first year postpartum [12]). Although there is notable heterogeneity in maternal depression trajectories [13], a 2017 systematic review highlighted two consistent patterns of symptoms across studies: a low-risk trajectory characterized by stable, subclinical depressive symptoms and a high-symptom trajectory characterized by persistent, clinically elevated depressive symptoms [14]. Compared to the vast literature on perinatal depression, less research has explored risk for perinatal anxiety; however, emerging research demonstrates elevated rates of maternal anxiety across pregnancy and postpartum [15]. Notably, despite research predominantly focusing on maternal well-being during the perinatal period, fathers are also at elevated risk for depression [16] and anxiety [17]. Indeed, evidence suggests that fathers experience similar rates of perinatal depression as mothers and that depressive symptoms remain relatively stable up to 2 years postpartum [18]. Given that maternal and paternal mental health can profoundly impact the family system [19], there is a critical need for research aimed at better understanding symptom trajectories among both parents across the perinatal period.

Although parents are at increased risk for depression and anxiety across the perinatal period, limited research has examined trajectories of broad-spectrum symptoms underlying both disorders. Instead, research has primarily focused on depression and anxiety as separate constructs despite substantial rates of comorbidity [20] and overlap in diagnostic criteria and symptoms [21, 22]. Further, given that symptoms of depression and anxiety may overlap with normal experiences that occur during and after pregnancy (e.g., fatigue and sleep disruption), it remains unclear whether mothers and fathers experience elevations in core internalizing symptoms (i.e., negative affectivity, the underlying feature across depression, and anxiety disorders) across the perinatal period.

The Hierarchical Taxonomy of Psychopathology (HiTOP [23]) addresses these limitations through its dimensional and hierarchical approach to psychopathology. Within this framework, symptoms that share similar features and tend to covary are grouped into syndromes (disorders), and correlated syndromes can be explained by overarching subfactors. These subfactors can be combined further into spectra, which represent the higher-order dimensions of psychopathology (e.g., internalizing, externalizing). Within the HiTOP model, the observed comorbidity between depression and anxiety reflects the broader internalizing spectrum [24, 25]. Importantly, the option to focus assessment at the level of the internalizing *spectrum* allows researchers and clinicians to account for the nonspecific emotional and cognitive symptoms of internalizing disorders, thus capturing the shared feature of negative affectivity [25, 26], rather than relying on assessment of specific *symptoms*, which may be confounded by the overlap between mood/anxiety symptoms and typical pregnancy-related experiences. Additionally, the HiTOP model emphasizes dimensional assessment of psychopathology, focusing on the level of severity rather than categorical diagnosis [23, 27]. Such dimensional assessment is especially important when considering the construct of psychopathology, which lacks clear divisions between levels [28], and permits examination of clinical information beyond the presence/absence of a disorder [29]. Nonetheless, the HiTOP model also recognizes the utility of such classifications and is designed to accommodate such practices (e.g., using clinically meaningful cutoff scores [30]). Finally, emerging research demonstrates that the HiTOP framework is applicable to pregnancy and that internalizing problems are central to psychopathology during this period [31]. Thus, research isolating changes in negative affectivity across the perinatal period is critical for understanding the risk for internalizing problems and has the potential to reveal transdiagnostic risk and protective factors.

Existing research has identified a range of sociodemographic, contextual, and psychological factors that appear to confer risk for elevated depression and anxiety symptoms across the perinatal period [13, 14, 15, 32, 33, 34]. For instance, mothers with a minoritized racial or ethnic background are at increased risk for depressive symptoms [35], and financial strain (i.e., low income and unemployment) is associated with maternal depression and anxiety [15, 35], as well as paternal depression [34]. Evidence also suggests that parents with children already in the home may experience more internalizing symptoms than parents experiencing the transition to parenthood for the first time [36].

Beyond these sociodemographic characteristics, stress is a robust predictor of maternal and paternal depression [34, 37, 38, 39, 40] and, to a lesser extent, maternal anxiety [15]. Concerns related to pregnancy and childbirth have been associated with elevated maternal depressive symptoms [38]. Further, a history of psychiatric problems (e.g., depression) has consistently been identified as a predictor of maternal perinatal depression and anxiety [13, 15, 37, 38, 40], as well as paternal postpartum depression [34]. Notably, maternal depression is also a risk factor for paternal perinatal depression [16, 34, 41]. Finally, contextual factors related to the intimate relationship, namely poorer relationship quality and lack of social support, have been linked to maternal depression and anxiety [13, 15, 40], as well as paternal depression [32, 34, 42].

Despite studies suggesting that these factors confer risk, Baron et al. [14] concluded in a systematic review that “none of the demographic, personality or clinical characteristics… systematically differentiated groups of women with different symptom trajectories, within or across studies” (p. 194). Notably, extant research has largely focused on risk factors, and less attention has been paid to protective factors that might reduce risk for perinatal depression and anxiety. Research employing a strengths-based, rather than problem-focused, approach to understanding internalizing problems across the perinatal period has important implications for prevention and early intervention efforts [43]. Specifically, focusing on *adaptive capacities* has the potential to identify pre-existing resources that can be harnessed or bolstered to promote mental health across pregnancy and the postpartum period. In particular, resources that help parents modulate negative emotions are expected to play a critical role in reducing the risk for internalizing problems across this transition.

A transdiagnostic factor in the development of internalizing problems and psychopathology, more broadly, is poor emotion regulation (i.e., difficulties managing the intensity and duration of emotions [44]). Importantly, a growing body of work suggests that effective emotion regulation can reduce the risk for internalizing symptomology [45]. Although extant research has primarily focused on specific emotion regulation strategies (e.g., cognitive reappraisal and rumination), there is clinical utility in exploring how broader regulatory processes mitigate risk for internalizing problems. For instance, psychological flexibility encompasses several regulatory abilities, including acceptance of emotional experiences, the ability for individuals to distance themselves from their thoughts, and being present and responding in a nonjudgmental manner to one's thoughts and feelings [46]. Psychological flexibility is a core process targeted in mindfulness-based interventions, such as Acceptance and Commitment Therapy (ACT [47]), which incorporates both mindfulness and acceptance strategies to promote psychological well-being and is an empirically-supported treatment for depression and anxiety [48]. Although not a formal component of ACT, self-compassion is closely linked to mindfulness but also reflects the ability to be kind towards oneself when experiencing difficulties and to recognize the shared nature of suffering (i.e., common humanity [49]). Thus, while mindfulness promotes attunement to emotional experiences, acknowledging one's emotional experiences in a compassionate manner might be key for fostering acceptance and promoting overall well-being. Interventions directly targeting self-compassion have emerged in recent years, including Compassion-Focused Therapy [50] and the Mindful Self-Compassion Program [51]. Given the clinical relevance of these constructs, research exploring the relative contributions of psychological flexibility and self-compassion to internalizing problems across the perinatal period has important implications for targeted prevention and intervention efforts during this unique transitional period.

Although most research on emotion regulation has focused on intrapersonal strategies, researchers increasingly recognize that social relationships also play a critical role in regulating emotional experiences [52]. Specifically, interpersonal resources (e.g., social support and attachment security) may be used to manage emotional arousal and expression, a process often referred to as *Interpersonal Emotion Regulation* [52, 53]. For individuals in committed relationships, intimate partners often represent a key resource for supporting emotion regulation and health. For couples navigating pregnancy, in particular, intimate partners can be critical sources of support given the shared experience, and promoting a high-quality interparental bond during pregnancy may serve as an important resource for navigating pregnancy stress [54, 55, 56] and mitigating risk for prenatal depression [57]. This work is consistent with the *Couple and Family Discord Model of Depression* and related research demonstrating that discord in intimate relationships poses a significant risk for depression [58, 59] and, to some degree, anxiety [60].

Examining how prenatal intrapersonal and interpersonal regulatory resources relate to the core features of internalizing disorders across the perinatal period is important for several reasons. First, the pregnancy-postpartum transition is a major life event during which mothers and fathers may rely more heavily on emotion regulation resources to manage increased levels of stress associated with this transition [61]. Second, mothers and their partners engage more regularly with the healthcare system during the perinatal period. As such, identifying whether specific regulatory processes uniquely impact risk for internalizing disorders has important implications for prevention and intervention efforts promoting individual and couple well-being. Finally, given that symptoms of depression and anxiety may overlap with common experiences that occur during and after pregnancy (e.g., fatigue, sleep disruption, weight changes), research focusing on broader affective symptoms has the potential to elucidate whether mothers and fathers experience elevations in core internalizing symptoms across the perinatal period.

The overarching aim of the present study was to examine trajectories of core internalizing symptoms spanning pregnancy to 2 years postpartum and to identify modifiable factors that confer risk for or protect against elevated psychopathology. Specifically, building upon past research and theory, we conducted a systematic investigation of (a) modifiable risk factors during pregnancy (i.e., concerns related to pregnancy and childbirth, partner psychopathology) and (b) transdiagnostic intrapersonal and interpersonal regulatory resources that might mitigate risk for internalizing problems across the perinatal period. After identifying significant correlates of symptom profiles, we sought to establish the directionality of effects by controlling for a history of internalizing problems. Finally, we examined significant predictors simultaneously to isolate specific factors that are most critical for promoting maternal and paternal well-being.

There are several innovative features of the present study. The present study is the first, to our knowledge, to examine trajectories of core internalizing symptoms among mothers and fathers using growth mixture modeling (GMM). Identifying trajectories of core internalizing symptoms that transcend both depression and anxiety—symptoms that are also not confounded with typical pregnancy-related experiences (e.g., sleep disruption)—has the potential to advance existing evidence-based interventions. Further, by embracing a strength-based approach and focusing on modifiable risk factors and pre-existing intrapersonal and interpersonal regulatory capacities, the present study has important implications for prevention efforts that may help couples cope with stressors associated with the pregnancy to postpartum transition. Lastly, by examining predictors of symptom profiles simultaneously, the present study has the potential to isolate specific factors that are most critical for promoting maternal and paternal well-being and contribute to the development of more targeted and efficient prevention and intervention efforts with transdiagnostic implications.

## 2. Materials and Methods

### 2.1. Participants and Procedure

All procedures were approved by the University of Nebraska-Lincoln Institutional Review Board. Participants were recruited via flyers and brochures that were broadly distributed to businesses and clinics frequented by pregnant women (e.g., obstetric clinics). We also established cooperative arrangements with multiple agencies in the community, and, if permitted, members of the research team approached potential participants to provide a brief overview of the study along with a brochure. Eligibility criteria included (a) 19 years of age or older (legal age of adulthood in Nebraska), (b) English speaking, (c) pregnant at the time of the initial appointment, (d) both biological parents of the child, (e) singleton pregnancy, and (f) in a committed intimate relationship and cohabiting. One hundred sixty-two mixed-sex couples enrolled in the study during pregnancy. Three couples were excluded from the final sample due to either ineligibility or invalid data, resulting in a sample of 159 couples (159 women and 159 men). Demographic characteristics are reported in Table 1.

The present study consisted of five waves of data collection spanning February 2016 to March 2020. At pregnancy (*M* = 28.03 weeks, SD = 7.75), both partners attended a laboratory appointment, for which they were each compensated $50, and completed self-report questionnaires and a semi-structured clinical interview assessing the quality of their intimate relationship. Partners completed these assessments in separate rooms and did not interact with one another until procedures were complete. At 1 month (*M* = 1.12 months, SD = 0.29), 6 months (*M* = 6.32 months, SD = 0.36), 1 year (*M* = 12.80 months, SD = 0.76), and 2 years postpartum (*M* = 24.50 months, SD = 0.66), each partner completed self-report questionnaires separately from one another. Each partner received $25 at the 1- and 6-month appointments and $100 at the 1- and 2-year appointments. At each timepoint, participants completed other procedures beyond the scope of the present study.

### 2.2. Measures

#### 2.2.1. Core Symptoms of Internalizing Disorders

Core symptoms of internalizing disorders were assessed using the Dysphoria subscale of the expanded version of the Inventory of Depression and Anxiety Symptoms (IDAS-II [62]). The IDAS-II is a self-report questionnaire comprised of 99 items to measure general and specific symptom dimensions of depression and anxiety-related disorders. Participants were asked to rate the extent to which they experienced each symptom over the previous 2 weeks on a five-point Likert scale from 1 (*not at all*) to 5 (*extremely*). The 10-item Dysphoria subscale captures nonspecific negative affectivity and measures key affective and cognitive symptoms underlying internalizing disorders [63]. The IDAS-II has demonstrated excellent psychometric properties in pregnant samples [64, 65], and the Dysphoria scale demonstrated good internal consistency across timepoints in the present study (*α*s ranging from 0.83 to 0.87).

#### 2.2.2. Modifiable Risk Factors

*(1) Concerns Related to Pregnancy and Childbirth*. Maternal concerns related to pregnancy and childbirth were assessed using the 10-item Pregnancy-Related Anxiety Scale (or Pregnancy-Related Thoughts Scale [66]). Mothers reported the extent to which they experienced concerns related to pregnancy, labor, and delivery (e.g., “I am afraid that I will be harmed during delivery”) over the past month on a scale ranging from 1 (*never/not at all*) to 4 (*a lot/very much*). Two items (“I am confident of having a normal childbirth” and “I think my labor and delivery will go normally”) were reverse-scored. Items were summed, with higher scores indicating elevated pregnancy-related concerns (*α =* 0.85).

*(2) Partner Internalizing Symptoms*. Partner internalizing symptoms during pregnancy were assessed using the Dysphoria subscale of the IDAS-II (see above) and explored as a predictor of symptom profiles (e.g., maternal dysphoria predicting paternal symptom profiles).

#### 2.2.3. Intrapersonal and Interpersonal Regulatory Resources

*(1) Psychological Flexibility*. Psychological flexibility was assessed using the Multidimensional Psychological Flexibility Inventory (MPFI [67]), a 60-item self-report measure that captures distinct dimensions of psychological flexibility and inflexibility. In the present study, participants were asked to respond to each item on a five-point scale (1 = *never true*, 2 = *rarely true*, 3 = *occasionally true*, 4 = *often true*, 5 = *always true*), whereas the published instrument uses a six-point scale that also includes a “very often true” response. Recent bifactor analyses suggest that the general flexibility and inflexibility scales are preferable to subscale scores, given that they are more stable and account for the most reliable item variance [68]. Thus, we computed a total score comprised of the 30 items reflecting the following dimensions of psychological flexibility: acceptance, present moment awareness, self as context, defusion, values, and committed action. Scores in the present study could range from 30 to 150 (*α* = 0.97), with higher scores indicating higher levels of psychological flexibility.

*(2) Compassionate Self-Responding*. Compassionate self-responding was measured using the Self-Compassion Scale (SCS [69]). The SCS is a 26-item self-report measure capturing dimensions of compassionate (e.g., “I try to be loving towards myself when I'm feeling emotional pain”) and uncompassionate self-responding (e.g., “I'm intolerant and impatient toward those aspects of my personality I don't like”). Participants were asked to respond to each item on a five-point scale ranging from 1 (*almost never*) to 5 (*almost always*). Consistent with factor analyses in past research [70], we computed a total score for compassionate nonresponding by aggregating items across the mindfulness, common humanity, and self-kindness subscales (four items each), with higher scores reflecting higher levels of compassionate self-responding (*α* = 0.89).

*(3) Indicators of Intimate Relationship Quality*. Facets of the intimate relationship were assessed using the Relationship Quality Interview (RQI [71, 72]), a 60-min semi-structured interview that captures dyadic functioning across multiple domains of the relationship over the past 6 months. *Emotional intimacy* assesses mutual closeness, warmth, interdependence, and affection. *Sexual quality* assesses satisfaction with the sexual relationship, including the presence/absence of negative emotions during sex and sexual difficulties. *Conflict management* assesses the frequency and length of arguments, as well as how couples resolve conflicts together. *Received support* assesses the quality of support received when the partner is feeling down or has a problem, including the extent to which there is a match between desired and received levels of support. Interviewers asked open-ended questions followed by close-ended questions to obtain contextual information and used concrete behavioral indicators to facilitate objective ratings of each domain on a scale ranging from 1 (*poor functioning*) to 9 (*high functioning*). Interviews with partners were completed simultaneously and were conducted and coded separately by different interviewers to prevent response contamination. The RQI has demonstrated excellent reliability and validity [71, 72]. In this sample, interrelater reliability was excellent (20% of cases; one-way random model, single rater intraclass correlation = 0.91). Consistent with recommended scoring procedures, both maternal and paternal interview scores were averaged to create dyadic scores of each domain except for support, as questions in this domain relate to the respondent's unique experiences receiving support from their partner (i.e., one partner might receive high-quality support while the other does not).

#### 2.2.4. Demographic Characteristics

Upon study entry, participants reported their race, ethnicity, annual joint income, and psychiatric history. Participants were categorized as having a minoritized racial/ethnic identity if they identified as non-White and/or Hispanic/Latino. After adjusting for household size, couples' annual household income was dichotomized based on the median household income in Nebraska at the time of study enrollment. Finally, participants who reported being diagnosed with or treated for depression, anxiety, or bipolar disorder were categorized as having a history of internalizing problems.

### 2.3. Data Analytic Plan

Data were analyzed using Mplus version 8.8 [73]. We used GMM [74] to identify unobserved subgroups of parents with distinct patterns of change in dysphoria over five repeated measures spanning pregnancy to 2 years postpartum. Separate models were tested for mothers and fathers. We estimated an intercept factor (i.e., symptom levels at childbirth) and a slope factor (i.e., annual rate of change), and factor variances were fixed to be equal within classes. We evaluated class enumeration using the Bayesian information criteria (BIC) and selected the model with the smallest BIC value that also produced substantively interpretable classes of adequate size. We also evaluated Entropy [75] to determine whether there was adequate class separation (values ideally >0.80 [76]). Finally, we conducted bootstrapped likelihood ratio tests (BLRT) to compare class solutions (e.g., 1-class versus 2-class). A significant BLRT suggests that the more complex solution, with more classes, is a better fit. After selecting the optimal number of classes, we applied a published cutoff score for the IDAS-II Dysphoria subscale for any internalizing diagnosis to characterize the classes clinically [30]. We selected the “balanced” cutoff score, which is optimal for discriminating between individuals who meet diagnostic criteria for any internalizing diagnosis and those who do not.

We also examined correlates of symptom profiles using the 3-step approach [77]. A correlate was considered significant if *p* < 0.05 and the 95% confidence interval did not include 1.00. A series of theoretically meaningful demographic characteristics associated with elevated internalizing symptoms across the perinatal period (i.e., minoritized racial/ethnic identity, low-income status, first-time parenthood) were explored as potential covariates. Once we identified significant correlates, we determined whether these factors remained significant after controlling for a history of internalizing problems to establish directionality. Finally, guided by these analyses, we examined whether significant predictors incrementally predicted class membership in an integrated model (i.e., a model with all significant predictors examined simultaneously).

### 2.4. Transparency and Openness

Data management and analysis procedures for this project, including the original power analysis guiding study design, were preregistered (https://osf.io/hprk8), and we made no deviations from this plan. Although participants did not consent to the open sharing of their raw data, Rebecca L. Brock (rebecca.brock@unl.edu) can be contacted to request access to research materials, analysis code, and data pending appropriate sharing approvals. Because we had prior knowledge of data from this longitudinal study, and given the exploratory nature of mixture models, we did not preregister study hypotheses.

## 3. Results

### 3.1. Profiles of Dysphoria Spanning Pregnancy to 2 Years Postpartum

**Figure 1:**
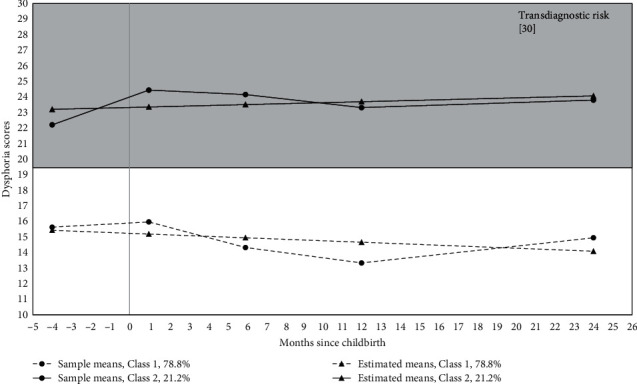
Maternal trajectories of core internalizing symptoms. *Note*. Class 1 = low symptoms, Class 2 = clinically elevated symptoms. Transdiagnostic risk was defined based on the screening cutoff score of the IDAS Dysphoria subscale for any internalizing diagnosis.

Descriptive statistics and correlations among study variables are reported in Table 2. For mothers, although a 3-class solution was the best fit to the data based on BIC values (1-class = 4,239.82; 2-class = 4,064.15; 3-class = 4,041.15), class separation was less than adequate (Entropy = 0.71). The 2-class solution had adequate class separation (Entropy = 0.86) and was a better fit than the 1-class solution (BLRT = 190.88, *p* < 0.001). In the 2-class solution, 21.2% of the mothers demonstrated elevated, fluctuating levels of dysphoria that exceeded the clinical threshold, whereas 78.8% of the mothers had relatively low levels of dysphoria that decreased at a significant rate over time (*b* = −0.05, *p*=0.006). See Figure 1 for a depiction of maternal symptom profiles from pregnancy to 2 years postpartum.

**Figure 2:**
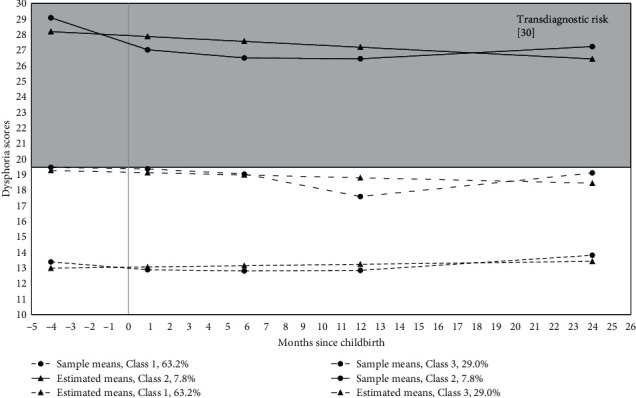
Paternal trajectories of core internalizing symptoms. *Note*. Class 1 = low symptoms, Class 2 = clinically elevated symptoms, Class 3 = subthreshold symptoms. Transdiagnostic risk was defined based on the screening cutoff score of the IDAS Dysphoria subscale for any internalizing diagnosis.

For fathers, a 3-class solution was the best fit to the data based on BIC values (1-class = 3,981.21; 2-class = 3,822.78; 3-class = 3,769.23; 4-class = 3,772.05). The 3-class solution had adequate class separation (Entropy = 0.84) and was a better fit than the 2-class solution (BLRT = 68.76, *p* < 0.001). In the 3-class solution, 7.8% of fathers demonstrated elevated, fluctuating levels of dysphoria, which exceeded the clinical threshold; 29.0% of fathers demonstrated “subthreshold” symptom levels (i.e., elevations that did not exceed the clinical cutoff but were near the screening threshold of 19.50), with symptoms fluctuating over time; and 63.2% of fathers had relatively low and stable levels of dysphoria. See Figure 2 for a depiction of paternal symptom profiles from pregnancy to 2 years postpartum.

### 3.2. Prenatal Correlates of Symptom Profiles

When predicting *maternal* dysphoria classes, only paternal (partner) dysphoria during pregnancy (OR = 1.11) and maternal concerns about pregnancy and childbirth (OR = 1.12) were significantly correlated with membership in the clinical profile. The remaining intra- and interpersonal factors were not significantly associated with class membership, nor were maternal demographic characteristics (i.e., minoritized racial/ethnic identity, low-income status, history of internalizing problems, first-time parenthood). See Table 3 for a summary of results.

When predicting *paternal* dysphoria classes, several intrapersonal and interpersonal factors were correlated with symptom profiles. Maternal (partner) dysphoria during pregnancy was significantly associated with membership in the clinical profile relative to the low symptom profile (OR = 1.14). Fathers with a history of internalizing problems were significantly more likely to belong to the clinical (OR = 110.11) and subthreshold (OR = 45.79) profiles relative to the low symptom profile. In contrast, fathers were significantly less likely to be in the clinical profile relative to the low symptom profile if they had higher levels of psychological flexibility (OR = 0.96), compassionate self-responding (OR = 0.52), emotional intimacy (OR = 0.42), or received support (OR = 0.41). Fathers with higher levels of emotional intimacy were also less likely to be in the subthreshold profile relative to the low symptom profile (OR = 0.57). Fathers were significantly less likely to be in the clinical profile relative to the subthreshold profile if they had higher levels of psychological flexibility (OR = 0.96). Sexual quality, conflict management, minoritized racial/ethnic identity, low-income status, and first-time parenthood were not significantly associated with class membership. See Table 4 for a summary of results.

### 3.3. Predictors of Profiles Controlling for History of Internalizing Problems

In the maternal models, after controlling for a maternal history of internalizing problems, paternal (partner) dysphoria during pregnancy (OR = 1.11) and maternal concerns about pregnancy and childbirth (OR = 1.12) significantly predicted maternal membership in the clinical profile relative to the profile characterized by low symptoms (Table 3).

In the paternal models, after controlling for a history of internalizing problems, compassionate self-responding and maternal (partner) dysphoria during pregnancy were no longer associated with class membership (Table 4). Demonstrating the protective role of regulatory resources, psychological flexibility uniquely predicted membership in the low symptom (OR = 0.96) and subthreshold profile (OR = 0.97) relative to the clinical profile. Similarly, received support uniquely predicted membership in the low symptom (OR = 0.38) and subthreshold profile (OR = 0.61) relative to the clinical profile. Emotional intimacy was uniquely associated with membership in low symptom profiles relative to the clinical (OR = 0.36) and subthreshold profiles (OR = 0.49).

### 3.4. Incremental Prediction of Profiles

Finally, we examined whether significant predictors of symptom profiles, as guided by the previous analyses, incrementally predicted class membership in an integrated model (i.e., a model with all predictors examined simultaneously). In the maternal model, when also controlling for a history of internalizing problems, both paternal (partner) dysphoria during pregnancy (OR = 1.12) and maternal concerns about pregnancy and childbirth (OR = 1.13) uniquely predicted membership in the clinical profile (Table 3).

In the paternal model, when also controlling for a history of internalizing problems, psychological flexibility, received support, and trust uniquely predicted class membership. Psychological flexibility uniquely predicted membership in the low symptom (OR = 0.95) and subthreshold (OR = 0.96) profiles relative to the clinical profile. Similarly, received support uniquely predicted membership in the low symptom (OR = 0.41) and subthreshold (OR = 0.53) profiles relative to the clinical profile. Interestingly, when controlling for psychological flexibility and received support, emotional intimacy uniquely predicted membership in the low symptom profile relative to the subthreshold symptom profile (OR = 0.50; Table 4).

## 4. Discussion

The present study is the first, to our knowledge, to investigate the risk for elevations in broad negative affectivity underlying depression and anxiety across the pregnancy-postpartum transition—unique from internalizing symptoms common to the perinatal period, such as sleep and appetite changes—among both mothers and fathers in committed intimate relationships. Further, the present study builds on extant research by examining modifiable risk and protective factors that can be mitigated or enhanced to reduce transdiagnostic risk for psychopathology across the perinatal period, which represents a critical opportunity for prevention and intervention.

Consistent with extant research on maternal depression [14], maternal internalizing problems manifested in two ways in the present study: a low-symptom profile characterized by symptoms that slowly decreased over time and a clinical profile characterized by clinically significant elevations in internalizing problems that, notably, persisted up to 2 years postpartum. Results converge with past research on rates of maternal depression (e.g., 23.8%; [78]) and suggest that approximately one in five mothers (21.2% in the present study) experience clinical elevations in broad internalizing problems across pregnancy and the postpartum period. Further, results also suggest that elevations in depressive and anxiety symptoms observed during this period can be attributed to core psychopathology rather than physical symptoms typically associated with the pregnancy-postpartum transition (e.g., appetite and sleep changes). Although fewer fathers experienced chronic, clinically significant elevations in internalizing problems (7.8%), we found that a substantial portion of fathers in the present study (29.0%) experienced subthreshold internalizing symptoms across the perinatal period, which still hold clinical significance. Interestingly, sample means for internalizing problems appeared to increase from 1 year to 2 years postpartum among both mothers in the low-symptom group and fathers in the low and subthreshold symptom groups. Although small in magnitude (i.e., 1–2 points on a scale with scores ranging from 10 to 50), these increases suggest that additional research exploring maternal and paternal internalizing symptoms beyond the first year postpartum is warranted.

We also identified unique and salient predictors of internalizing symptom profiles for both mothers and fathers. Mothers who reported more concerns related to pregnancy and childbirth (e.g., worries about their infant's health, fear of difficult labor/delivery) and whose partners reported elevated internalizing problems during pregnancy were significantly more likely to demonstrate a profile characterized by clinically significant elevations in internalizing problems, suggesting that these represent important targets for early intervention. Interestingly, mothers with a history of internalizing problems were not more likely to demonstrate clinically elevated internalizing symptoms across pregnancy and the postpartum. However, this is consistent with past research demonstrating that pregnancy represents a period of risk for both recurrent and new-onset psychopathology [79].

Regarding intrapersonal regulatory resources, neither maternal psychological flexibility nor self-compassion were significantly associated with class membership. One potential explanation is that these processes buffer the risk for maternal internalizing problems only in the context of stress or adversity (i.e., interaction vs. main effect), and future research exploring this possibility is warranted before more definitive interpretations can be made. Similarly, there were no significant associations between facets of the intimate relationship and maternal internalizing problems. This finding contrasts with a robust body of research demonstrating an association between couple discord and depression (refer to [59] for a review). It may be that, despite the importance of the intimate relationship, mothers do not rely exclusively on their partners for support and emotional intimacy and draw on other close relationships within their social networks [80] to promote well-being across the pregnancy-postpartum transition.

Although not associated with maternal symptom profiles, intrapersonal and interpersonal regulatory processes emerged as robust protective factors against elevated *paternal* internalizing problems across pregnancy and the postpartum period. Controlling for other significant predictors and a history of internalizing problems, paternal psychological flexibility uniquely predicted membership in the low and subthreshold symptom profiles relative to the clinically elevated symptom profile. This finding is consistent with research demonstrating that effective emotion regulation reduces the risk for internalizing problems [45]; further, it suggests that fathers' abilities to employ regulatory strategies in response to stressors in a flexible and nonjudgmental manner may be particularly important across the transition from pregnancy to postpartum. Perhaps psychological flexibility is uniquely protective for fathers because, compared to mothers, they lack tailored support to cope with stress and the competing demands of fatherhood (e.g., work vs. caregiving [81]). Indeed, given research that fathers experience barriers to seeking both formal and informal support [82], pre-existing regulatory abilities may be particularly important across pregnancy and the postpartum period.

Controlling for psychological flexibility, emotional intimacy, and a history of internalizing problems, the quality of received support in the intimate relationship uniquely predicted membership in the low and subthreshold symptom profiles relative to the clinically elevated symptom profile. Further, when controlling for other significant predictors, emotional intimacy predicted membership in the low symptom profile relative to the subthreshold symptom profile but *not* relative to the clinically elevated symptom profile. These findings align with research demonstrating an association between partner support and postpartum depression [83] and are particularly notable given that the quality of the intimate relationship emerged as protective for fathers but not for mothers. Research suggests that fathers often feel invisible across the perinatal period [84]. Specifically, fathers may believe that their primary role is to be strong, available, and regulated for their partner and fear judgment by medical professionals when they experience emotional challenges [84]. Further, as previously noted, fathers experience difficulties accessing support; therefore, emotional intimacy and high-quality support may be uniquely protective for fathers, as they are less likely to seek support outside of the interparental relationship.

Finally, maternal internalizing symptoms during pregnancy were not associated with elevated paternal internalizing problems across the perinatal period. This finding contrasts with past research suggesting that maternal depression confers risk for paternal depression [16]; however, it is possible that maternal internalizing problems confer risk through other, more proximal processes related to the quality of the intimate relationship (e.g., support [83]). For instance, mothers who experience elevations in core internalizing symptoms may experience difficulties connecting emotionally with their partners and providing high-quality support, thereby impacting paternal wellbeing.

## 5. Implications

The results of the present study have important implications for prevention and early intervention efforts to promote parental well-being across the perinatal period. First, results highlight the importance of repeated screening for internalizing problems during pregnancy and up to 2 years postpartum. Although the American College of Obstetricians and Gynecologists [85] recommends that all women be screened for depression and anxiety during pregnancy and the postpartum period, screening is not implemented universally [86], particularly for anxiety disorders [87]. Further, extant research suggests that current screening tools for perinatal anxiety do not reliably or accurately identify women in need of further assessment and treatment [88]. The Dysphoria subscale of the IDAS-II, which captures broad liability for internalizing disorders, could be implemented as a brief screening tool, and women who exceed the established cutoff scores [30] could be referred to mental health providers for a more thorough diagnostic assessment to inform subsequent intervention efforts. In addition to improving screening practices, interdisciplinary efforts to improve medical providers' abilities to identify perinatal internalizing problems are critical given that mothers interact with multiple providers across pregnancy and the postpartum (e.g., obstetricians, family medicine practitioners, pediatricians), many of whom may lack knowledge or specific training in mental health [89]. Models of collaborative care that integrate behavioral health providers (e.g., social workers, psychiatric nurse practitioners, and psychologists) into medical practice may offer unique advantages by not only improving screening rates [90] but also providing increased access to accurate, sensitive assessment and the timely implementation of evidence-based interventions [91].

Notably, existing screening and intervention efforts often focus solely on mothers and overlook fathers. Results of the present study underscore the critical importance of routine prenatal and postnatal screening for fathers, as well as specific intervention efforts for fathers who report elevated symptoms. Our findings demonstrate a notable portion of fathers experience subthreshold levels of internalizing problems, which might be overlooked despite the fact that subthreshold symptoms may be associated with maladaptive functioning [92]. Indeed, the present study demonstrated that paternal internalizing symptoms impact not only fathers, but also maternal mental health spanning pregnancy to 2 years postpartum. Given that fathers may be less likely to vocalize distress due to beliefs that their needs are secondary to mother and baby during the perinatal period [84], it is vital that medical professionals conduct routine mental health screening with fathers and create a nonjudgmental space for fathers to discuss emotional difficulties with the aim of encouraging fathers to access resources. Importantly, fathers are not always present during routine appointments, and outreach efforts that provide assessment and resources to fathers may be beneficial across this unique transition.

Our findings also indicated that mothers who endorsed concerns related to pregnancy and childbirth—concerns that are distinct from general worry and anxiety [93]—were more likely to exhibit elevated internalizing symptoms across the perinatal period, even when controlling for past internalizing problems. This suggests that providing mothers with effective coping skills to manage pregnancy concerns during the prenatal period may reduce the risk of internalizing symptoms in the postpartum period. Thus, in addition to assessing internalizing problems more broadly, it may be beneficial for providers to implement brief screening measures for pregnancy-specific concerns, such as the Pregnancy-Related Anxiety Questionnaire-Revised [94] or the Childbirth Fear Questionnaire [95], during routine prenatal visits. Treatment recommendations relevant to perinatal women include evidence-based transdiagnostic interventions for depression and anxiety, such as Cognitive Behavioral Therapy [96] and ACT [97], which could also be implemented to address unique concerns related to pregnancy (i.e., fear of childbirth [98]) while also promoting emotion regulation skills. Additionally, psychoeducation about the specific changes that occur during the perinatal period (e.g., hormonal fluctuations that can increase the risk for mood disturbances) might help normalize mothers' emotional experiences, as well as specific pregnancy-related concerns. The implementation of perinatal-specific assessment tools coupled with early intervention efforts has the potential to prevent the negative implications of maternal perinatal depression and anxiety to the larger family system (e.g., interparental relationship issues, bonding difficulties, infant development).

Results of the present study also build on the *Couple and Family Discord Model of Depression* by illuminating specific facets of the intimate relationship that appear to be particularly important for promoting paternal well-being across the transition from pregnancy to postpartum. By embracing a multifaceted conceptualization of intimate relationship quality, we identified the quality of received support and emotional intimacy as key protective factors for fathers. These findings converge with existing research demonstrating the efficacy of couples-based interventions for depression and relationship distress [59] and suggest that, beyond reducing relationship distress, fostering emotional intimacy and enhancing the *quality* as opposed to the mere *frequency* of support are especially important for paternal wellbeing. In particular, results point towards the utility of the Marriage Checkup (MC), an evidence- and strengths-based two-session intervention with foundations in Integrative Behavioral Couple Therapy that was designed to prevent the emergence of serious relationship concerns (e.g., aggression in conflict) and reduce barriers to engaging in traditional couples therapy (e.g., extensive time and financial commitments [99]). Importantly, the MC has demonstrated efficacy in reducing both relationship distress and depressive symptoms [100] and is not limited to use with married couples [101]. Further, the MC was recently adapted to prevent perinatal mood and anxiety and piloted as the Before Baby Relationship Checkup to couples in an obstetric clinic, with preliminary results demonstrating its feasibility and acceptability [102]. Thus, both the MC and the Before Baby Relationship Checkup hold promise for promoting well-being across the transition from pregnancy to postpartum, particularly for individuals experiencing subthreshold levels of distress that may not otherwise engage in treatment. Indeed, given that paternal internalizing problems emerged as a key predictor of elevated maternal symptoms, improving key components of the couple's relationship has the potential to improve not only paternal outcomes but also maternal outcomes and downstream consequences (e.g., better family functioning and child outcomes).

Finally, given the protective role of psychological flexibility, fathers who report clinically elevated internalizing problems during pregnancy may benefit from individual or group-based mindfulness-based interventions (e.g., mindfulness-based childbirth and parenting programs, ACT). Though most work examining the effectiveness of these interventions during the perinatal period focuses on mothers [97, 103], mindfulness-based interventions across the perinatal period demonstrate preliminary feasibility and acceptability among fathers [104] and have been shown to improve paternal mindfulness and reduce paternal internalizing symptoms [105]. Importantly, because many parents already seek childbirth education during pregnancy and pregnant women prefer mindfulness-based interventions to other treatment approaches [106], mindfulness-based childbirth and parenting programs that incorporate both mothers and fathers may be particularly appealing. Further, several brief adaptations of mindfulness-based childbirth and parenting programs exist (e.g., a weekend workshop, 4 vs. 9 weeks [105, 107]), thereby enhancing the accessibility of these interventions.

## 6. Limitations and Future Directions

The results of the present study should be viewed in light of several limitations. Study aims were pursued in a community sample with relatively low levels of psychopathology, and results may not generalize to at-risk couples experiencing higher levels of psychopathology. Our sample also consisted of mixed-sex pregnant couples who were both biological parents of the child (for aims beyond the scope of the present study) and who identified as being in a committed intimate relationship and cohabiting. As such, the results of the present study may not apply to all birthing and non-birthing parents. Exploring patterns and predictors of internalizing problems among a more diverse group of parents is particularly important given that sexual and gender minority parents are at greater risk for internalizing problems [108] and may experience stigma and discrimination in obstetrical settings [109]. Relatedly, representative of the region where the study was conducted, participants predominantly identified as White, non-Hispanic/Latino, and middle-class, which limits the generalizability of results. Although minoritized racial/ethnic identity was not associated with symptom profiles in the present study, future research exploring the impact of minority stress on perinatal mental health with a more diverse sample is critical given the well-documented racial and ethnic disparities in internalizing problems across the perinatal period [110]. We also did not assess the role of culture or geographic background, which represents an important direction for future research. The present study was also limited to one prenatal assessment that occurred at varying times depending on study enrollment, and additional work is needed to pinpoint the ideal timeframe for screening and early intervention efforts. Finally, the present study did not clarify the relationship between trajectories of parental internalizing problems and long-term family and child outcomes, which represents an important direction for future investigation.

## 7. Conclusion

In conclusion, the results of the present study demonstrate that a substantial portion of mothers and fathers in community samples may experience elevations in internalizing symptoms during pregnancy that persist up to 2 years postpartum. We found that mothers' pregnancy-related concerns and fathers' internalizing symptoms during pregnancy conferred risk for clinically elevated maternal internalizing problems across the perinatal period. In contrast, emotional intimacy, the quality of received support, and psychological flexibility emerged as key protective factors for fathers, suggesting the potential efficacy of couples-based interventions during the perinatal period to improve relationship quality and subsequent mental health outcomes, as well as mindfulness-based interventions to improve intrapersonal emotion regulation. Overall, findings underscore the importance of enhancing screening practices during pregnancy and the postpartum period, expanding intervention efforts to include fathers, and enhancing the intimate relationship quality to buffer risk for internalizing problems across this critical family transition.

## Figures and Tables

**Table 1:** Demographic characteristics (*N* = 159 couples; 318 individuals).

Demographic characteristics	Maternal	Paternal
Parental characteristics at study entry	Mean (SD) or % (*n*)	

Age at study entry	28.67 (4.27)	30.56 (4.52)
Race and ethnicity		
Non-Hispanic/Latino, white	83.6% (133)	85.5% (136)
Hispanic/Latino, white	5.7% (9)	1.9% (3)
American Indian/Alaskan Native	0.6% (1)	5.7% (9)
Asian	2.5% (4)	2.5% (4)
Black or African American	0.6% (1)	3.8% (6)
More than one race	6.9% (11)	0.6% (1)
Education		
Did not complete high school	1.9% (3)	1.9% (3)
GED	1.3% (2)	1.9% (3)
High school diploma	3.8% (6)	6.9% (11)
Vocational, technical, or associate's degree	6.3% (10)	13.2% (21)
Some college	13.8% (22)	17.0% (27)
Bachelor's degree	46.5% (74)	34.6% (55)
Master's degree	19.5% (31)	14.5% (23)
Doctorate	6.9% (11)	10.1% (16)
Employed	74.2% (118)	91.8% (146)

Family characteristics at study entry	Mean (SD) or % (*n*)	

Length of relationship (months)	81.90 (49.59)	
Length of cohabitation (months)	61.00 (41.80)	
Number of children	0.79 (1.18)	
First-time parents	57.9% (92)	
Married	84.9% (135)	
Low-income status	49.1% (78)	

*Note*. Approximately one in four couples included a partner who identified as an ethnic or racial minority, with many couples (15.1% of the sample) representing multi-racial households.

**Table 2:** Descriptive statistics and correlations.

Dysphoria scores	1	2	3	4	5	6	7	8	9	10
1. T1 maternal dysphoria	1.00	—	—	—	—	—	—	—	—	—
2. T2 maternal dysphoria	0.46^*∗∗∗*^	1.00	—	—	—	—	—	—	—	—
3. T3 maternal dysphoria	0.49^*∗∗∗*^	0.49^*∗∗∗*^	1.00	—	—	—	—	—	—	—
4. T4 maternal dysphoria	0.56^*∗∗∗*^	0.43^*∗∗∗*^	0.68^*∗∗∗*^	1.00	—	—	—	—	—	—
5. T5 maternal dysphoria	0.45^*∗∗∗*^	0.45^*∗∗∗*^	0.48^*∗∗∗*^	0.63^*∗∗∗*^	1.00	—	—	—	—	—
6. T1 paternal dysphoria	0.25^*∗∗*^	0.22^*∗∗*^	0.21^*∗∗*^	0.26^*∗∗*^	0.19^*∗*^	1.00	—	—	—	—
7. T2 paternal dysphoria	0.15	0.42^*∗∗∗*^	0.19^*∗*^	0.11	0.23^*∗∗*^	0.57^*∗∗∗*^	1.00	—	—	—
8. T3 paternal dysphoria	0.15	0.25^*∗∗*^	0.34^*∗∗∗*^	0.25^*∗∗*^	0.18^*∗*^	0.46^*∗∗∗*^	0.47^*∗∗∗*^	1.00	—	—
9. T4 paternal dysphoria	0.17^*∗*^	0.20^*∗*^	0.21^*∗∗*^	0.19^*∗*^	0.21^*∗∗*^	0.55^*∗∗∗*^	0.48^*∗∗∗*^	0.43^*∗∗∗*^	1.00	—
10. T5 paternal dysphoria	0.19^*∗*^	0.34^*∗∗∗*^	0.14	0.19^*∗*^	0.17^*∗*^	0.73^*∗∗∗*^	0.50^*∗∗∗*^	0.48^*∗∗∗*^	0.59^*∗∗∗*^	1.00
Mean	17.03	17.74	16.29	15.39	16.81	16.37	15.69	15.45	15.20	16.37
SD	5.06	6.21	5.86	5.29	5.63	5.82	5.83	5.69	5.24	5.18
*N*	159	142	133	119	117	159	128	119	113	112

*Note*. T1 = pregnancy, T2 = 1 month postpartum, T3 = 6 months postpartum, T4 = 1 year postpartum, T5 = 2 years postpartum. *⁣*^*∗*^*p* < 0.05, *⁣*^*∗∗*^*p* < 0.01, *⁣*^*∗∗∗*^*p* < 0.001.

**Table 3:** Correlates and predictors of maternal symptom profiles.

Risk and protective factors	Clinical vs. low		
	OR	95% CI	*p*-Value
Correlates of symptom profiles			

*Modifiable risk factors*			
Pregnancy-related anxiety	**1.12**	**[1.03, 1.22]**	**0.010**
Paternal (partner) dysphoria	**1.11**	**[1.04, 1.19]**	**0.003**
*Intrapersonal protective factors*			
Psychological flexibility	*0.97*	[*0.95*, *1.00*]	*0.064*
Compassionate self-responding	0.65	[0.35, 1.22]	0.183
*Interpersonal protective factors*			
Emotional intimacy	0.69	[0.43, 1.10]	0.114
Sexual quality	*0.73*	[*0.53*, *1.01*]	*0.060*
Conflict management	*0.71*	[*0.49*, *1.03*]	*0.073*
Quality of received support	0.90	[0.65, 1.25]	0.530
*Demographic characteristics*			
Minoritized racial/ethnic identity	0.45	[0.11, 1.92]	0.281
Low-income status	1.15	[0.49, 2.73]	0.751
History of internalizing problems	2.11	[0.78, 5.70]	0.141
First-time parenthood	1.47	[0.60, 3.60]	0.405

Predictors controlling for history of internalizing problems			

Pregnancy-related anxiety	**1.12**	**[1.02, 1.22]**	**0.017**
Paternal (partner) dysphoria	**1.11**	**[1.04, 1.18]**	**0.003**

Incremental prediction			

Pregnancy-related anxiety	**1.13**	**[1.03, 1.24]**	**0.009**
Paternal (partner) dysphoria	**1.12**	**[1.03, 1.22]**	**0.009**

*Note*. OR, odds ratio. Significant effects (*p* < 0.05) are bolded. Marginal effects (*p* < 0.10) are italicized.

**Table 4:** Correlates and predictors of paternal symptom profiles.

Risk and protective factors	Clinical vs. subthreshold			Clinical vs. low			Subthreshold vs. low		
	OR	95% CI	*p*- Value	OR	95% CI	*p*- Value	OR	95% CI	*p*- Value
Correlates of symptom profiles									

*Modifiable risk factors*									
Maternal (partner) dysphoria	1.07	[0.94, 1.21]	0.342	**1.14**	**[1.002, 1.29]**	**0.047**	1.07	[0.99, 1.16]	0.109
*Intrapersonal protective factors*									
Psychological flexibility	**0.96**	**[0.93, 0.99]**	**0.005**	**0.96**	**[0.92, 0.99]**	**0.011**	1.00	[0.97, 1.02]	0.755
Compassionate self-responding	*0.58*	[*0.32*, *1.05*]	*0.070*	**0.52**	**[0.30, 0.90]**	**0.020**	0.90	[0.52, 1.56]	0.700
*Interpersonal protective factors*									
Emotional intimacy	0.74	[0.35, 1.57]	0.436	**0.42**	**[0.21, 0.85]**	**0.015**	**0.57**	**[0.33, 0.98]**	**0.042**
Sexual quality	1.12	[0.66, 1.90]	0.670	0.84	[0.52, 1.34]	0.458	*0.75*	[*0.53*, *1.05*]	*0.092*
Conflict management	0.74	[0.40, 1.37]	0.329	*0.61*	[*0.35*, *1.07*]	*0.083*	0.83	[0.58, 1.19]	0.318
Quality of received support	*0.65*	[*0.40*, *1.04*]	*0.073*	**0.41**	**[0.23, 0.74]**	**0.003**	*0.63*	[*0.39*, *1.04*]	*0.071*
*Demographic characteristics*									
Minoritized racial/ethnic identity	3.18	[0.60, 16.94]	0.176	2.79	[0.70, 11.20]	0.148	0.88	[0.26, 3.00]	0.836
Low-income status	*2.22*	[*0.94*, *5.24*]	*0.070*	0.66	[0.14, 3.10]	0.597	*0.30*	[*0.07*, *1.19*]	*0.087*
History of internalizing problems	2.41	[0.56, 10.27]	0.236	**110.11**	**[3.50, 3467.71]**	**0.008**	**45.79**	**[1.41, 1486.88]**	**0.031**
First-time parenthood	1.79	[0.41, 7.82]	0.929	1.06	[0.28, 3.99]	0.439	0.59	[0.26, 1.38]	0.227

Predictors controlling for history of internalizing problems									

Maternal (partner) dysphoria	1.07	[0.95, 1.20]	0.284	*1.13*	[*0.98*, *1.30*]	*0.083*	1.06	[0.96, 1.16]	0.234
Psychological flexibility	**0.97**	**[0.94, 0.996]**	**0.026**	**0.95**	**[0.91, 0.99]**	**0.024**	0.98	[0.94, 1.02]	0.289
Compassionate self-responding	0.59	[0.30, 1.16]	0.128	*0.56*	[*0.28*, *1.09*]	*0.089*	0.94	[0.53, 1.69]	0.841
Emotional intimacy	0.75	[0.38, 1.47]	0.398	**0.36**	**[0.17, 0.77]**	**0.008**	**0.49**	**[0.26, 0.90]**	**0.022**
Quality of received support	**0.61**	**[0.38, 0.98]**	**0.042**	**0.38**	**[0.21, 0.69]**	**0.001**	*0.62*	[*0.37*, *1.05*]	*0.075*

Incremental prediction									

Psychological flexibility	**0.96**	**[0.93, 0.99]**	**0.006**	**0.95**	**[0.92, 0.98]**	**0.002**	0.99	[0.96, 1.01]	0.317
Emotional intimacy	1.36	[0.66, 2.78]	0.404	0.68	[0.29, 1.56]	0.358	**0.50**	**[0.26, 0.97]**	**0.041**
Quality of received support	**0.53**	**[0.30, 0.94]**	**0.029**	**0.41**	**[0.23, 0.73]**	**0.003**	0.79	[0.50, 1.24]	0.304

*Note*. OR, odds ratio. Significant effects (*p* < 0.05) are bolded. Marginal effects (*p* < 0.10) are italicized.

## Data Availability

Although participants did not consent to the open sharing of their raw data, Rebecca L. Brock (rebecca.brock@unl.edu) can be contacted to request access to research materials, analysis code, and data pending appropriate sharing approvals.

## Additional Points

*Author Note*. This study complied with Transparency and Openness Promotion (TOP) Guidelines. PI Rebecca L. Brock (rebecca.brock@unl.edu) should be contacted to request access to research materials, analysis code, and data. Data management and analysis procedures were preregistered (https://osf.io/hprk8), and we made no deviations from that plan. Because we had prior knowledge of data from this study, we did not preregister hypotheses. Data from this sample have been published elsewhere [64, 111, 112]; however, this is the first article to explore trajectories of internalizing symptoms across pregnancy and postpartum and to identify predictors of these trajectories.
